# Effects of Summer and Autumn Drought on Eutrophication and the Phytoplankton Community in Dongting Lake in 2022

**DOI:** 10.3390/toxics11100822

**Published:** 2023-09-29

**Authors:** Guanghan Yan, Xueyan Yin, Xing Wang, Yunyu Zhang, Enrui Wang, Zhibing Yu, Xingliang Ma, Minsheng Huang

**Affiliations:** 1 State Environmental Protection Key Laboratory of Drinking Water Source Protection, Chinese Research Academy of Environmental Sciences, Beijing 100012, China; yangh012@163.com (G.Y.); yinxueyu@126.com (X.Y.); 13681339599@163.com (E.W.); 2 National Engineering Laboratory for Lake Pollution Control and Ecological Restoration, Chinese Research Academy of Environmental Sciences, Beijing 100012, China; 3 State Environmental Protection Scientific Observation and Research Station for Lake Dongting, Chinese Research Academy of Environmental Sciences, Beijing 100012, China; 4 School of Ecological and Environmental Sciences, East China Normal University, Shanghai 200241, China; 15882287931@163.com; 5 Hunan East Dongting Lake National Nature Reserve, Yueyang 414000, China; zhibinyu2023@163.com (Z.Y.); 19918300018@163.com (X.M.)

**Keywords:** Dongting Lake, eutrophication, environmental factors, phytoplankton, hydrological connectivity

## Abstract

Since July 2022, the Yangtze River basin has experienced the most severe hydro-meteorological drought since record collection started in 1961, which has greatly affected the ecological environment of the Dongting Lake (DTL) basin. To investigate the effects of drought events on the eutrophication and phytoplankton community structure of DTL, the lake was sampled twice in August and September 2022 based on the water level fluctuations resulting in 47 samples. Furthermore, we combined the comprehensive trophic level index (TLI) and phytoplankton Shannon–Wiener diversity index (*H*) to characterize and evaluate the eutrophication status. The key influencing factors of the phytoplankton community were identified using redundancy analysis (RDA), hierarchical partitioning, and the Jaccard similarity index (*J*). Our results showed that the TLI of DTL changed from light–moderate eutrophication status (August) to mesotrophic status (September), whereas the *H* changed from light or no pollution to medium pollution. The phytoplankton abundance in August (122.06 × 10^4^ cells/L) was less than that in September (351.18 × 10^4^ cells/L) in DTL. A trend in phytoplankton community succession from Bacillariophyta to Chlorophyta and Cyanophyta was shown. The combination of physiochemical and ecological assessment more accurately characterized the true eutrophic status of the aquatic ecosystem. The RDA showed that the key influencing factors in the phytoplankton community were water temperature (WT), pH, nitrogen and phosphorus nutrients, and the permanganate index (COD_Mn_) in August, while dissolved oxygen (DO) and redox potential (ORP) were the key factors in September. Hierarchical partitioning further indicated that temporal and spatial variations had a greater impact on the phytoplankton community. And the *J* of each region was slightly similar and very dissimilar, from August to September, which indicated a decreased hydrological connectivity of DTL during drought. These analyses indicated that the risk to the water ecology of DTL intensified during the summer–autumn drought in 2022. Safeguarding hydrological connectivity in the DTL region is a prerequisite for promoting energy flow, material cycle, and water ecosystem health.

## 1. Introduction

Lakes play an important role in regulating ecological balance at global and regional scales, mainly by maintaining biodiversity, purifying water bodies, and regulating local climate [1,2,3]. However, lakes are becoming increasingly threatened by agricultural modernization, industrialization, and urbanization [4,5,6]. Meanwhile, climate change is also having an impact on lake ecosystems, especially climate extremes (i.e., floods and droughts) [7,8]. For a long time, the instability of the monsoon affected droughts in China [9,10,11]. Extreme drought events cause a series of negative environmental and ecological effects, such as water scarcity, water quality deterioration, and reduced aquatic biodiversity [12,13,14]. As an important primary producer of water ecosystems, phytoplankton is very sensitive to changes in the water environment [15,16]. Their species and abundance are affected by various abiotic (i.e., N and P nutrients, light conditions, water temperature, hydrological connectivity, etc.) [17,18,19] and biotic (i.e., zooplankton) factors [20]. Therefore, phytoplankton is considered to be indicator organisms for the evaluation of water quality and ecological quality of rivers and lakes [21,22].

Specifically, drought events affect aquatic ecosystems by prolonging hydraulic residence time, altering water transport patterns in the catchment, and changing internal processes (i.e., photosynthesis and respiration) [12,23]. Changes in water from short river flows to prolonged evaporation and high residence time have a significant impact on phytoplankton biomass and trophic dynamics, which are characterized by phytoplankton blooms during water scarcity seasons as well as pronounced water acidification and eutrophication [12,14]. Previous studies endeavored to unravel the dominant effects of droughts on water quality constituents in inland waters, while most determinants (including water temperature, dissolved oxygen, pH, salinity, major ions, nutrients, organic carbon, and metals) showed mixed responses to droughts [24,25]. In addition, Wang et al. showed that the extreme drought event in 2022 led to significant warming of surface water temperatures in lakes in southwest China and the Yangtze River Basin, which in turn may lead to an increase in thermal stratification, a decrease in water level, and a decrease in surface area and dissolved oxygen, among other changes in the physicochemical properties of water [26]. This will undoubtedly have a great impact on water eutrophication and water ecosystems.

Dongting Lake is the second largest freshwater lake in China, and the first large-scale river-connected lake in the middle and lower reaches of the Yangtze River from the Three Gorges Dam (TGD) [27], which receives water from the three channels and the four rivers [28]. The composition of the lake follows seasonal patterns in dry and flood seasons with fluctuating water levels [29,30]. Meanwhile, the water surface slope shows a decreasing trend in the dry season except in the flood season (July to September) [31]. However, since the flood season in 2022, under the influence of the continuous La Niña event, the Yangtze River Basin has experienced the most serious meteorological and hydrological drought since 1961, with nearly 50% less rainfall and a continued low water level of the Yangtze River mainstream [9]. The water level of the mainstream of the Yangtze River continues to be low, which in turn caused the cut-off of the Yangtze River entering the Dongting Lake in September, resulting in a rapid shrinkage of the watershed area, dry bottom, and narrowed living space for aquatic organisms. At present, investigations of the extreme drought events in Dongting Lake mainly focuses on the hydrometeorological analysis of the causes of drought [32,33,34] and the impact on fishery farming and wetlands [35,36]. However, there is no study on the impacts of extreme hydrometeorological drought on the aquatic ecosystem of the lake area. It is critical to determine the eutrophication status and phytoplankton community structure changes affected by the extreme drought events to better understand the driving mechanism of lake aquatic systems.

In this study, we investigated the water quality and phytoplankton community structure of the Dongting Lake Basin under successive drought events in the summer and autumn of 2022. The purpose of this study was to (1) analyze the effects of extreme drought events on eutrophication in Dongting Lake; (2) study the effects of extreme drought events on the phytoplankton community structure in Dongting Lake; and (3) explore the effects of hydrological connectivity on the hydroecological environment by combining the diversity index and the similarity index in Dongting Lake. These data can be used to mitigate the impact of drought on water ecology and ensure the water ecological security of Dongting Lake.

## 2. Materials and Methods

### 2.1. Study Area

**Figure 1:**
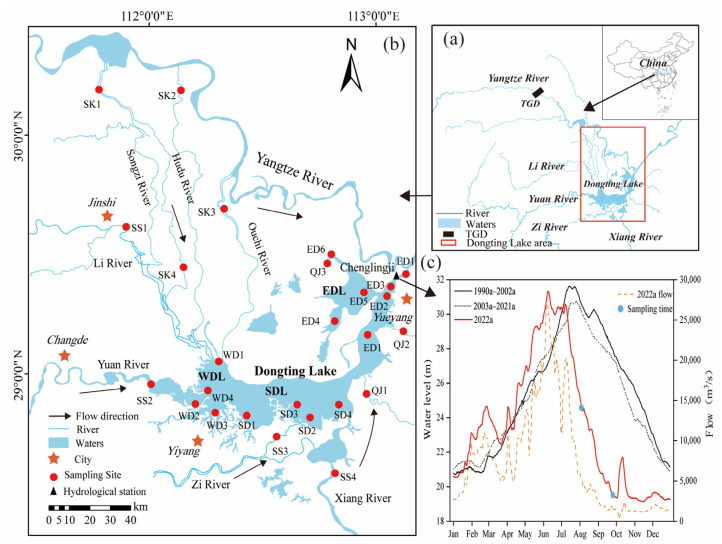
(**a**) Water system in Dongting Lake. (**b**) Sampling sites in Dongting Lake. (**c**) Annual water level variation in Chenglingji Hydrographic Station at different stages (Jan, January; Feb, February; Mar, March; Apr, April; May, May; Jun, June; Jul, July; Aug, August; Sep, September; Oct, October; Nov, November; Dec, December).

Dongting Lake (110°40′~113°40’ E, 28°30′~30°20’ N) is located in the northern part of Hunan Province, on the south bank of the middle reaches of the Yangtze River (Figure 1a). The drainage area is 2625 km^2^, the volume is 1.78 × 1010 m^3^, and the average water depth is 6.39 m [37]. The water level and water storage in the flood season are 1.14–1.64 and 17–31 times that in the dry season, respectively [38]. Dongting Lake consists of West Dongting Lake (WDL, 284 km^2^), South Dongting Lake (SDL, 897 km^2^), and East Dongting Lake (EDL, 1217 km^2^). The WDL receives incoming water from the Songzi, Taiping, and Ouchi branches of the Jingjiang River of the Yangtze River and the Yuan and Li Rivers. The SDL receives water from the Zi River and Xiang River, and the EDL receives water from the Xinwall River, Miluo River, and Huarong River. Water from the WDL and SDL flows into the Yangtze River from Chenglingji through the EDL, forming a complex river-flowing lake [39].

### 2.2. Sampling

A total of 26 sampling sites were set up in the whole basin, and the sampling sites were divided into 6 groups according to the distribution and environmental differences in the sampling sites: SK1–SK4, representing the incoming water from three channels of the Yangtze River; SS1–SS4, representing the four rivers including Li River, Yuan River, Zi River and Xiang River; QJ1–QJ3, representing the interval incoming water of the lake district; WD1-WD4, representing the WDL; SD1–SD4, representing the SDL; and ED1–ED7, representing the EDL (Figure 1b). Compared with 1990–2002a and 2003–2021a, the water level at Chenglingji Hydrological Station from January to June 2022 was higher than that during the same period. However, the water level from July to December was significantly lower than in the same period due to the severe reduction in rainfall and water inflow in the Yangtze River Basin (Figure 1c). The summer and fall (July–September) are sensitive periods for algal blooms in Dongting Lake [28,40]. Therefore, samples were collected during August (hydrologically connected period) and September (disconnected period) in 2022, when the water level at Chenglingji Hydrographic Station was 24.57 m and 19.48 m, respectively (Figure 1c). The data on water level and discharge were derived from the Hunan Hydrology and Water Resources Survey Center (http://slt.hunan.gov.cn/, accessed on 20 June 2023). Notably, due to the low water level in September, the sampling points QJ3, WD4, SD3, ED4, and ED5 showed a large area of beach exposure, so samples could not be collected. In total, 47 samples were collected in this study.

### 2.3. Sample Analysis

Water temperature (WT, °C), dissolved oxygen (DO, mg/L), electrical conductivity (EC, μS/cm), turbidity (Turb, NTU), total dissolved solids (TDS, mg/L), the oxidation-reduction potential (ORP, mV), and pH value were obtained using YSI-EXO professional plus in situ. The water transparency (SD, m) was measured using the Secchi disk method.

Nutrient analysis was measured within 24 hours following Chinese national standards [41]. Total nitrogen (TN) was measured using the alkaline potassium persulfate digestion-UV spectrophotometric method, ammonia nitrogen (NH_4_^+^-N) was determined using Nessler’s reagent spectrophotometric method, nitrate (NO_3_^−^-N) was determined using ultraviolet spectrophotometry, total phosphorus (TP, mg/L) and orthophosphate (PO_4_^3−^-P) were determined using the ammonium molybdate method, the permanganate index (COD_Mn_) was determined using the acidic potassium permanganate method, dissolved silicate (DSi) was determined using the silicon molybdenum yellow method, total organic carbon (TOC) was analyzed using a total organic carbon analyzer (OI Analytical, 1030 W), and total suspended solids (TSS) was determined using the 105 °C drying weight method. These measurements were completed within 24 hours of sampling.

In the meantime, water within the 0.5 m depth from the surface was treated as surface water, which was collected in a 1 L plastic bottle and fixed with 15 mL Lugo’s iodine solution immediately. After the samples were kept in the laboratory for 48 h, they were concentrated to 30 mL. Phytoplankton cells were counted using an Olympus biomicroscope (CX31). A counting frame of 0.1 mL was used, which contained 100 horizons at 10 × 40 magnification [41]. Taxa were classified and identified to the genus level according to Hu and Wei [42].

### 2.4. Data Analysis

Following the nutritional status classification from the Organization for Economic Cooperation and Development (OECD), we used the comprehensive trophic level index (TLI) formula:

(1) $$\mathrm{TLI}\;(\Sigma )=\sum_{j=1}^{m}W_{j}{TLI}_{j}$$

where TLI (Σ) is the comprehensive trophic level index, W*j* is the weight of the trophic level index of the *j*th parameter, and TLI*j* is the trophic level index of the *j*th parameter. The TLI parameter includes: Chl-a, TP, TN, SD, and COD_Mn_.

The dominant species of phytoplankton were determined based on the dominance value *Y* for each species [43], phytoplankton diversity was represented with the Shannon–Wiener index (*H*) [44], and similarity of phytoplankton communities in different regions was calculated using the Jaccard’s similarity coefficient (*J*) [45], as follows:

*Y* = (*n_i_*/*N*)·*f_i_* (2)

(3) $$H=-\sum P_{i}\log_{2}P_{i}$$

*J* = *c*/(*a* + *b* − *c*) (4)

where *P_i_* = *N_i_*/*N*, *N_i_* is the number of species *i*, *N* is the total number observed, *n_i_* is the total number of individuals of species *i*, and *f_i_* is the frequency of occurrence in the sample. When *Y* > 0.02, the corresponding species is regarded as the dominant species. *a* is the number of species in water area A, *b* is the number of species in water area B, and *c* is the number of species shared between the two waters. The Shannon–Wiener index and the Jaccard similarity principle are generally divided into 4 and 6 levels, respectively [46,47], and the specific classification standards are shown in Table 1.

The significant differences in environmental factors in Dongting Lake were analyzed using one-way ANOVA and SPSS 21.0 statistical software. First, based on the analysis results for DCA, when the eigenvector (DCA1) of the first axis of DCA was less than 3, RDA analysis was selected. When DCA1 was between 3 and 4, both RDA and CCA were selected. When DCA1 was greater than 4, CCA was selected.

The variance inflation factor (VIF) was used to select environmental factors less than 20 for the redundancy analysis (RDA), which was used to evaluate the effects of environmental factors on phytoplankton communities. RDA and hierarchical segmentation analysis were performed using the rdacca.hp package [48]. The “envfit” function in the vegan package was used to test the significance of environmental factors affecting phytoplankton communities. Finally, the corrplot package was used to analyze the correlation between environmental factors and the Shannon–Wiener index. The above analysis process and visualization were completed in R (v.4.0.3).

## 3. Results

### 3.1. Environmental Parameter Variation and Eutrophication State

#### 3.1.1. Environment Parameter Variation

**Figure 2:**
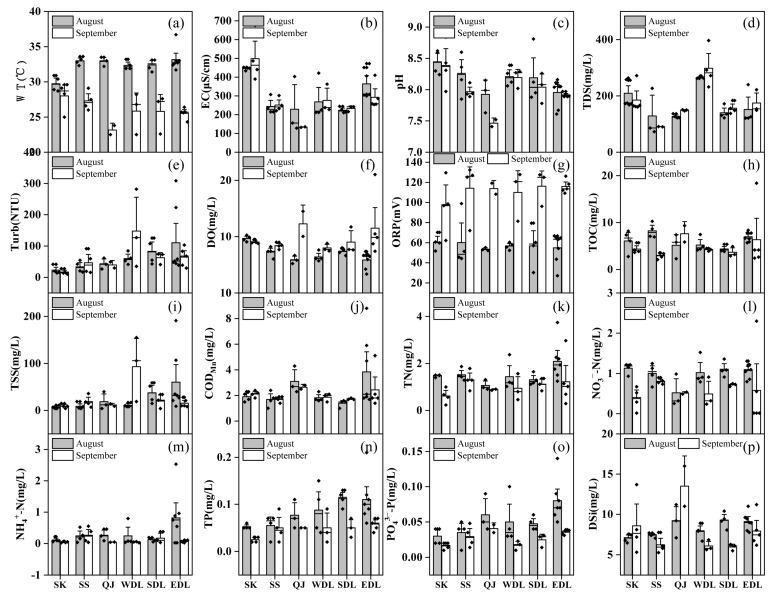
Spatiotemporal distribution of physicochemical properties in each region of Dongting Lake. (**a**) WT, water temperature; (**b**) EC, electrical conductivity; (**c**) pH, pH value; (**d**) TDS, total dissolved solids; (**e**) Turb, turbidity; (**f**) DO, dissolved oxygen; (**g**) ORP, the oxidation-reduction potential; (**h**) TOC, total organic carbon; (**i**) TSS, total suspended solids; (**j**) COD_Mn_, the permanganate index; (**k**) TN, total nitrogen; (**l**) NO_3_^−^-N, nitrate; (**m**) NH_4_^+^-N, ammonia nitrogen; (**n**) TP, total phosphorus; (**o**) PO_4_^3−^-P; (**p**) DSi, dissolved silicate. The bars represent the mean value; the error lines represent the standard deviation between the sampling sites in that region; and the small dots represent the value at each sampling site.

The one-way ANOVA results showed that WT, DO, ORP, TN, NO_3_^−^-N, NH_4_^+^-N, TP, and PO_4_^3−^-P were significantly different between August and September (*p* < 0.05) (Figure 2a,f,g,k–o). WT ranged from 22.5 °C to 36.7 °C, and WT in August (32.1 °C) was significantly higher than that in September (26.3 °C). In contrast, DO and ORP were significantly higher in September (DO = 9.60 mg/L, ORP = 110.19 mv) than in August (DO = 7.06 mg/L, ORP = 57.37 mv). For nutrients, TN and TP concentrations were significantly higher in August (TN = 1.58 mg/L, TP= 0.08 mg/L) than in September (TN = 1.02 mg/L, TP = 0.04 mg/L). Meanwhile, TN and TP concentrations were much higher than those of 0.2 mg/L and 0.02 mg/L, respectively. For spatial distribution, EC, pH, TDS, Turb, COD_Mn_, TP, PO_4_^3−^-P, and DSi were significantly different (*p* < 0.05) (Figure 2b–e,j,n,o,p). Among them, the EC and pH values of SK were the highest, and the QJ was the lowest. The TDS and Turb were the highest in WDL, and the Turb of the lake was significantly higher than that of the other three regions. The COD_Mn_ and phosphorus nutrient in EDL were significantly higher than in other areas. The DSi was highest in SK, followed by EDL.

#### 3.1.2. Eutrophication State

**Figure 3:**
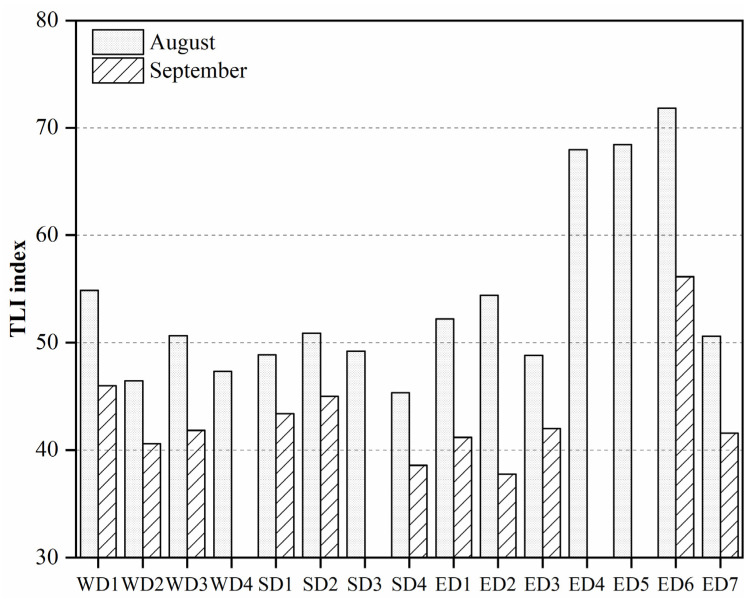
Temporal and spatial distribution of the TLI index at different sampling points in Dongting Lake.

The TLI index was between 37.74 and 71.83, with an average of 49.30 in DTL. According to the evaluation criteria of the TLI index, the DTL was in a mild eutrophication state in August (Figure 3), with EDL (59.18) > WDL (49.81) > SDL (48.57). The mesotrophic state was found in September, with EDL (43.82) > WDL (42.80) > SDL (42.32). Notably, ED4 (67.95), ED5 (68.43), and ED6 (71.83) of EDL reached a moderate to severe eutrophication state in August, and ED6 was still in a mild eutrophication state in September.

### 3.2. Distribution Characteristics of the Phytoplankton Community in Dongting Lake

#### 3.2.1. Phytoplankton Community Composition

**Figure 4:**
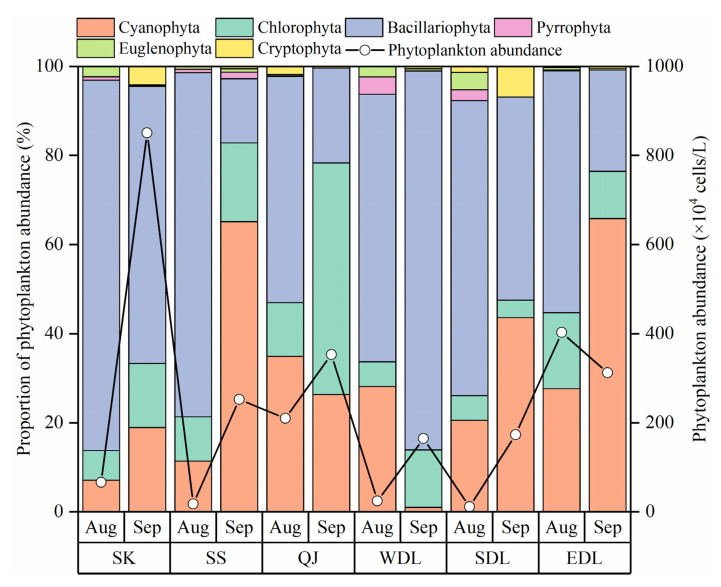
Spatial and temporal distribution of phytoplankton species proportion and abundance in Dongting Lake (Aug, August; Sep, September).

A total of 151 species of phytoplankton belonging to six phyla and 77 genera were identified in August and September in DTL, among which Bacillariophyta (39.1%), Chlorophyta (31.8%), and Cyanophyta (18.5%) were abundant. The abundance of phytoplankton ranged from 11.56 to 851.06 × 10^4^ cells/L in the entire basin, with an average density of 236.62 × 10^4^ cells/L (Figure 4). The abundance of phytoplankton in August (122.06 × 10^4^ cells/L) was less than in September (351.18 × 10^4^ cells/L). In August, the abundance of phytoplankton was the highest in EDL, followed by QJ, SK, WDL, SS, and SDL. In September, the abundance of phytoplankton was the highest in SK, followed by QJ, EDL, SS, SDL, and WDL.

A comparison of the percentage of phytoplankton abundance phyla in August and September indicated that except for WDL, all regions showed an increasing trend in Cyanophyta and Chlorophyta and a decreasing trend in Bacillariophyta in September, especially in SS, QJ, SDL, and EDL, where the changes were more obvious (Figure 4).

#### 3.2.2. Dominant Species of Phytoplankton

The results of dominance (*Y*) in Table 2 showed that there were 23 species of phytoplankton in three phyla in DTL. The number of dominant species in August was higher than in September. In August, the dominant species were *Synedra* sp., *Navicula* sp., *Nitzschia* sp., *Melosira granulata*, *Melosira granulata* var. *angutissima*, *Cyclotella* sp., and *Pseudanabaena* sp. In September, the dominant species were *Melosira granulata*, *Melosira granulata* var. *angutissima*, *Cyclotella* sp., *Phormidium tenue*, *Microcystis* sp., and *Merismopedia tenuissima*. In addition, the dominant species were Cyanophyta and Chlorophyta in QJ in September, and Cyanophyta was dominated in SDL and EDL.

#### 3.2.3. Phytoplankton Diversity (*H*) and the Community Similarity Index (*J*)

**Figure 5:**
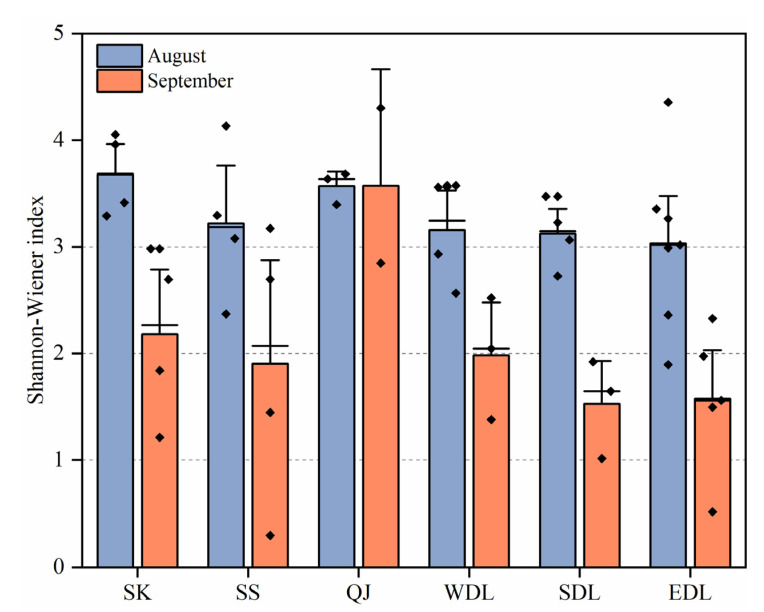
Spatiotemporal distribution of the Shannon–Wiener index at different sampling sites in Dongting Lake. The bars represent the mean value; the error lines represent the standard deviation between the sampling sites in that region; and the small dots represent the value in each sampling site.

During the survey period, the Shannon–Wiener index of phytoplankton in DTL ranged from 1.53 to 3.67, with an average value of 2.71 (Figure 5), indicating moderate pollution; its value was higher in August (3.30) than in September (2.12). In terms of regions, EDL showed the lowest average value (2.31), followed by SDL (2.32), SS (2.56), WDL (2.57), SK (2.93), and QJ (3.57). It is worth noting that in August, the Shannon–Wiener index was greater than 3 in all regions, suggesting each was in a light-pollution or pollution-free state. However, the overall region was between 1 and 2 in September, suggesting it was in a medium-pollution state. The lake area was more polluted than QJ and SS.

Based on the results of the similarity analysis (Table 3), the phytoplankton community similarity indexes of each region were in Grade III and Grade II during August and September, respectively, which indicated slightly similar levels and very dissimilar levels, respectively. In addition, EDL and QJ were in Grade IV and Grade III during August and September, respectively, which indicated moderately similar and slightly similar levels.

### 3.3. Driving Factors of the Phytoplankton Community Structure

**Figure 6:**
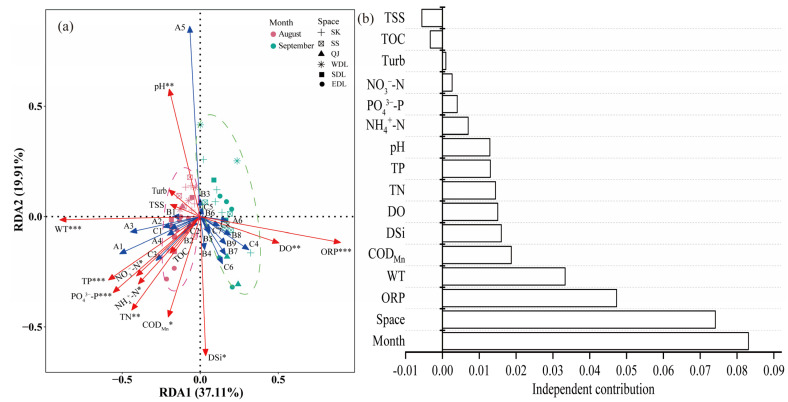
RDA (**a**) and hierarchical partitioning analysis (**b**) of the phytoplankton community and environmental factors in Dongting Lake. The environmental factors with *, **, and *** indicate *p* < 0.05, *p* < 0.01, and *p* < 0.001, respectively, which calculated using the “envfit” function test. The red arrow represents the environmental factors, the blue arrow represents the dominant species. The pink dashed-line circle and green dashed-line circle represent the quadrat distribution in different regions of Dongting Lake in August and September, respectively.

Based on the results of DCA (DCA1 = 2.14), the cumulative explanation of RDA for environmental factors on the first two axes of phytoplankton was 57.02% (Figure 6a). Their distributional relationships showed that the August and September sampling sites were concentrated in quadrants two/three and one/four, respectively, with high monthly similarity. Among them, August was mainly influenced by WT, pH, nitrogen and phosphorus nutrients, and COD_Mn_; September was mainly influenced by DO and ORP. *Synedra* sp., *Nitzschia* sp., *Melosira granulata*, and *Pseudanabaena* sp. were positively correlated with WT, nitrogen and phosphorus nutrients, and COD_Mn_. *Melosira granulata* var. *angutissima* was positively correlated with pH and negatively correlated with DSi. *Crucigenia rectangularis*, *Eudorina* sp., *Pediastrum simplex*, and *Phormidium tenue* were positively correlated with DO and ORP.

Hierarchical partitioning further showed that monthly variation in the physicochemical indicators affected the phytoplankton community more than spatial regions and that differences in spatial physicochemical indicators also significantly affected the phytoplankton community structure (Figure 6b). Moreover, the physicochemical factors that significantly affected the structure of the phytoplankton community (*p* < 0.05) were ORP, which was the highest, followed by WT, COD_Mn_, DSi, DO, and nitrogen and phosphorus nutrients.

**Figure 7:**
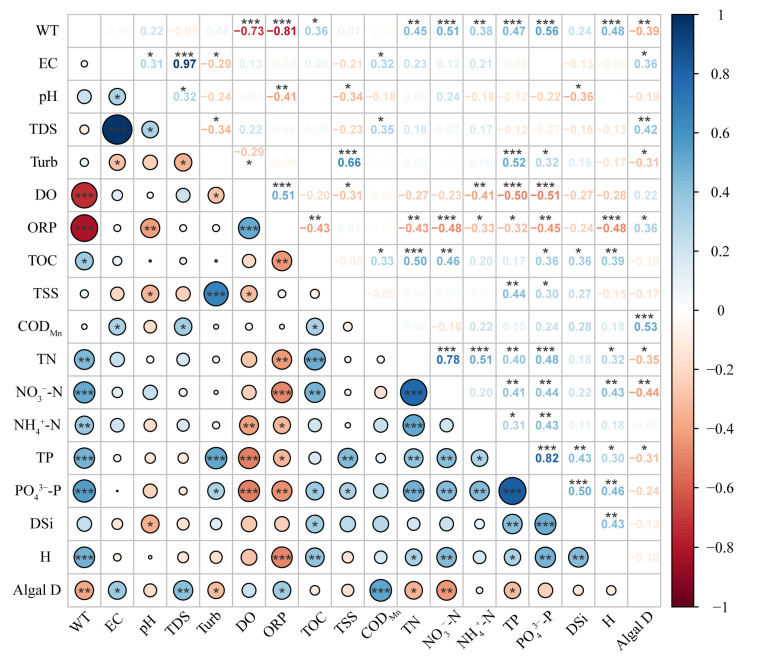
Correlation analysis between environmental factors and phytoplankton in Dongting Lake. ***, **, and * indicate significant correlations at the 0.001, 0.01, and 0.05 levels, respectively.

Further, the Spearman correlation analysis showed (Figure 7) that WT was significantly negatively correlated with DO and ORP and positively correlated with nitrogen and phosphorus nutrients (*p* < 0.01). Phytoplankton abundance was negatively correlated with WT, Turb, TN, NO_3_^−^-N, and TP and positively correlated with ORP and COD_Mn_ (*p* < 0.01). The Shannon–Wiener index was positively correlated with WT and nutrients (*p* < 0.01).

### 3.4. Comparison with Other Freshwater Lakes

A comparison of the differences in phytoplankton abundance and eutrophication status before and after the drought in DTL and in other freshwater lakes in the middle and lower reaches of the Yangtze River showed that the phytoplankton abundance in this study was much higher than that in DTL from 1991 to 2020 (10.0—95.7 × 10^4^ cells/L) (Table 4). Compared with the same time period in DTL and Poyang Lake, August was lower than the same time period in the two lakes in 2017, and September was much higher than the same time period in DTL in 2019. For the Shannon-Wiener index, the value in this study was higher than 3 in August than in Poyang Lake in August 2017 and Chaohu Lake in October 2020, which was higher than in the same period in 2017 in DTL, while in September, the value was lower than the same period in 2019 in DTL. For the TLI index, the mean value in this study in August was not much different from the same period in DTL in 2017, Chaohu Lake, and Hongze Lake, and in some areas, it is higher than the same period in 2017 in DTL, but much higher than that of DTL (1991–2020).

It is worth mentioning that the changes in phytoplankton abundance, the Shannon–Wiener index, and the TLI index in different studies were not exactly the same, which could cause variation in the characterization of the true eutrophication state of the water body if the TLI index were used as a single factor to evaluate the eutrophication status.

## 4. Discussion

### 4.1. Integrated Characterization of Eutrophication in Dongting Lake under Drought Events

During the drought period, DTL was in light eutrophic and mesotrophic states in August and September, respectively, and the eutrophication status of EDL was the most serious, reaching moderate–heavy eutrophication. Compared with the results of the previous research on DTL in the same period of the year, especially in the past 30 years (Table 4) [49,50], the eutrophication degree of DTL caused by drought events increased significantly. There might be two main reasons: (1) The water quality of DTL is greatly affected by the large number of nutrients carried by the three channels of the Yangtze River, the four rivers, and the incoming water from the shores [54]. However, the dryness of the Yangtze River Basin increased in mid-July, and it entered into the dry period in early August [55]. As a result, the reduction in the incoming water from the Basin weakened the diluting effect of the water body, which led to increased eutrophication in August. (2) The more heavily polluted water by agricultural practices in the DTL basin and rainfall runoff increased pollutants in the lake area [56]. As the drought intensified, the three channels of the Jingjiang River went dry one after another in September, and the little rainfall in the first half of September [55] reduced the surface runoff pollution. Therefore, the degree of eutrophication in September was improved. Because EDL is the convergence area of pollutants coming from WDL and SDL, the most serious eutrophication occurred in EDL. The Big-small West Lake at the end of the backwater of EDL was still mildly eutrophic in September, mainly due to the isolation of its water body since September, and the eutrophic state of its water body was already higher in August.

Lake eutrophication status can be more accurately assessed by combining physicochemical measurements including nitrogen, phosphorus, and other environmental factors with an analysis of primary producers including higher aquatic plants or phytoplankton [16,57]. The Shannon–Wiener index indicated all areas of DTL were in a light or non-polluted state and a medium-polluted state in August and September, respectively. The pollution state of the lake was higher than that of the incoming water (Figure 5), which showed an opposite trend to the results of the TLI index. A similar result was also observed in a study of Chaohu Lake (Table 4) [52], which is likely due to the fact that changes in phytoplankton habitat during the drought period led to changes in community diversity. Connell proposed the moderate disturbance hypothesis in 1978, which suggests that species diversity is maximized at medium intensity and frequency of disturbances at a specific time scale, and species diversity is maintained at a lower level at lower or higher intensities and frequencies [58]. At the beginning of the drought in August, the water level in Chenglingji was 24.57 m. At this time, the hydrological and hydrodynamic conditions were more suitable for the growth of most algae, and it was difficult to form absolute dominant species. In September, the water level dropped to 19.48 m (Figure 1c), and the hydrological connection of the lake was blocked, forming several blocked lakes with shallow water depths, which resulted in a lower diversity index under stronger wind disturbances or lower disturbances. Therefore, a decrease in the diversity index also implied an increase in the density of dominant algal species and even the risk of algal bloom outbreaks.

In general, the combination of physiochemical and ecological assessment can more accurately characterize the true eutrophic status of a water body. Moreover, due to the accumulation of surface pollution during the drought, the heavy precipitation process after drought causes a large amount of surface pollutants to be flushed into the water body, which may have a prominent effect on the eutrophic status.

### 4.2. Response of Phytoplankton Communities to Environmental Factors during Drought Events

In this study, nitrogen and phosphorus nutrient concentrations (Figure 2k–o) and TLI indexes (Figure 3) were higher in August than in September, while the abundance of phytoplankton showed the opposite trend. Moreover, the phytoplankton abundance showed an evolutionary trend from Bacillariophyta to Cyanophyta and Chlorophyta, especially in SS, QJ, SDL, and EDL (Figure 4). Drought caused varying degrees of impact on the hydrological characteristics and physicochemical factors of water bodies. It has been shown that drought-induced water level reduction is an important driver of phytoplankton dynamics, and lowering water level changes the light and mixing state, increases phytoplankton biomass, and favors cyanobacterial blooms [59,60,61]. Diatoms have a thick shell, settle easily, and need a certain flow rate to help with photosynthesis [62], which allowed them to be dominant in August. The nitrogen and phosphorus nutrient concentrations and TLI index improved in September, but its nutrient concentration was still higher than the threshold required for phytoplankton growth, and the falling water level formed a still lake that provided a good habitat for phytoplankton growth, which was especially suitable for the growth of Cyanophyta and Chlorophyta [63].

In addition, the RDA showed that the key phytoplankton influencing factors were WT, pH, nitrogen and phosphorus nutrients, and COD_Mn_ in August, and DO and ORP in September, which was consistent with a study on the phytoplankton in Danjiangkou Reservoir [64]. The hierarchical partitioning analysis further showed that temporal and spatial variations had a greater impact on the phytoplankton community, suggesting that changes in hydrological connectivity caused greater habitat heterogeneity related to spatial environmental factors (Figure 6b). ORP, WT, COD_Mn_, DSi, and DO were the main physicochemical influencing factors. WT is an important factor influencing the composition of phytoplankton communities, which can affect enzyme activities, nutrient uptake rates, and the cell cycle to varying degrees by altering the metabolic rate of phytoplankton cells [65]. Wang et al. demonstrated that an extreme high-temperature event in the summer of 2022 led to unprecedented warming of Chinese lakes, with an increase of 1.63 °C in the average surface WT of lakes, which was much higher than the observed surface WT in the past 20 years [26]. The direct result of this extreme increase in WT (i.e., above normal) was the catastrophic mortality of fish, benthic organisms, and aquatic plants [66]. Meanwhile, changes in WT can cause changes in physicochemical properties such as ORP, pH, DO, and nutrients. For instance, an increase in WT decreases DO and deoxygenation occurs in lakes, leading to the release of nutrients from lake sediments and accelerated eutrophication [67]. A significant negative correlation between WT and DO and a significant positive correlation between nitrogen and phosphorus nutrients were confirmed (Figure 7). These factors further affected the phytoplankton community, resulting in a lower abundance of phytoplankton in August and a higher abundance in September.

It is worth noting that the dominant species including *Synedra* sp., *Navicula* sp., *Nitzschia* sp., *Melosira granulata*, *Melosira granulata* var. *angutissima*, *Cyclotella* sp., and *Pseudanabaena* sp. were eutrophic taxa tolerant to turbidity and low-light, confirming the typical environment of Dongting Lake in August. And the increase in the number of dominant species of Cyanophyta in September, such as *Phormidium tenue*, *Microcystis* sp., and *Merismopedia tenuissima*, which exbibit fouling-tolerance, also implied that the continuous drought led to further intensification of the risk of hydro-ecology. Thus, the change in hydrological connectivity caused by drought events coupled with persistently high temperatures is an important factor affecting the phytoplankton community in DTL.

### 4.3. Effects of Lake Hydrologic Connectivity on Phytoplankton Community Heterogeneity during Drought Events

Hydrological flows act as carriers and media for nutrients required for the growth of aquatic organisms and transmit information on aquatic ecosystem succession [68]. A study of river-connected lakes (Lake Poyang) showed that hydrological connectivity changed the water exchange time and water flow velocity in the lake area, which led to significant changes in phytoplankton communities [69,70]. Therefore, phytoplankton communities in freshwater ecosystems with good hydrological connectivity tend to be similar [19]. In contrast, phytoplankton communities are less similar in aquatic habitats with little or no hydrologic connectivity [71]. Drought can lead to changes in horizontal and vertical hydrological processes, which in turn affect the flow of matter and energy in aquatic ecosystems [55]. In this study, the similarity indexes of phytoplankton communities in each region in August and September were at the levels of III and II, respectively (Table 3), i.e., mildly similar and very dissimilar, respectively. This result is consistent with the results of a study on phytoplankton in the arid zone of the Yellow River Basin [72].

Hydrologic and hydrodynamic conditions are the primary factors affecting the structure of phytoplankton communities in rivers and lakes [73]. Therefore, safeguarding hydrologic connectivity is a prerequisite for promoting energy flow, material cycling, and the health of aquatic ecosystems.

## 5. Conclusions

During the summer–autumn drought in 2022, the TLI index of DTL changed from light–moderate eutrophication status (August) to mesotrophic status (September), whereas the Shannon–Wiener index changed from light or no pollution to medium pollution. Thus, the combination of physiochemical and ecological assessment can more accurately characterize the true eutrophic status of the aquatic ecosystem. Moreover, the key factors influencing phytoplankton growth were WT, pH, nitrogen and phosphorus nutrients, and COD_Mn_ in August and DO and ORP in September. Overall, spatiotemporal variation had a greater impact on the phytoplankton community. Finally, we concluded that the similarity indexes of phytoplankton communities in each region in August and September were at the levels of mildly similar and very dissimilar, respectively, mainly due to changes in the hydrological connectivity of DTL. Thus, ensuring hydrological connectivity is a prerequisite for promoting energy flow, material cycling, and the health of aquatic ecosystems. In summary, the summer–autumn drought in 2022 resulted in increased WT and altered hydrological connectivity, which in turn led to a significant increase in the eutrophication problem of DTL and increased risk to the aquatic ecosystems. More attention should be paid to the response of lake aquatic ecosystems to extreme events. In future studies, high-frequency, multi-factor monitoring should be carried out to quantify the impacts of extreme events on lake aquatic ecosystems.

## Figures and Tables

**Table 1:** The criteria for the classification of the Shannon–Wiener and Jaccard similarity indexes.

Classification	Shannon–Wiener Index (*H*)		Jaccard Similarity Index (*J*)	
	Range	Description	Range	Description
I	0–1	Heavy pollution	0	No similar
II	1–2	α-Medium pollution	0.01–0.25	Highly dissimilar
III	2–3	β-Medium pollution	0.26–0.50	Slightly similar
IV	>3	Light or no pollution	0.51–0.75	Moderate similarity
V			0.76–0.99	Highly similar
VI			1	Completely similar

**Table 2:** Distribution of dominant species of phytoplankton in Dongting Lake.

Phyla	Code	Species	SK		SS		QJ		WDL		SDL		EDL	
			August	September	August	September	August	September	August	September	August	September	August	September
Bacillariophyta	A1	*Synedra* sp.	0.115	-	0.107	-	0.147	-	-	-	0.097	-	0.243	-
	A2	*Navicula* sp.	0.049	-	0.022	-	0.047	-	-	-	0.060	-	-	-
	A3	*Nitzschia* sp.	0.130	0.022	0.049	-	0.119	-	0.106	-	0.026	-	0.104	-
	A4	*Melosira granulata*	0.060	-	0.241	0.027	0.057	-	0.344	0.034	0.199	0.131	-	0.025
	A5	*Melosira granulata* var. *angutissima*	0.167	0.252	0.191	-	-	0.036	0.199	0.471	0.043	-	-	0.059
	A6	*Cyclotella* sp.	0.126	0.046	-	0.027	-	0.051	0.026	-	-	-	-	0.027
Chlorophyta	B1	*Chlorella* sp.	-	-	-	-	0.022	-	-	-	-	-	-	-
	B2	*Ankistrodesmus acicularis*	-	-	-	-	0.022	-	-	-	-	-	-	-
	B3	*Ulothrix* sp.	-	-	-	-	-	-	-	0.024	-	-	-	-
	B4	*Scenedesmus quadricauda*	-	-	-	-	0.035	0.116	-	-	-	-	-	-
	B5	*Scenedesmus bicaudatus*	-	-	-	-	-	0.029	-	-	-	-	-	-
	B6	*Crucigenia tetrapedia*	-	-	-	-	-	0.029	-	-	-	-	-	-
	B7	*Crucigenia rectangularis*	-	-	-	-	-	0.029	-	-	-	-	-	-
	B8	*Eudorina* sp.	-	-	-	0.053	-	0.029	-	-	-	-	-	-
	B9	*Pediastrum simplex*	-	-	-	0.023	-	0.029	-	-	-	-	-	-
Cyanophyta	C1	*Pseudanabaena* sp.	0.037	-	0.024	-	0.041	-	0.087	-	0.035	0.034	-	-
	C2	*Raphidiopsis* sp.	-	-	-	-	0.029	-	-	-	-	-	-	-
	C3	*Planktothricoides*	-	-	-	-	0.035	-	-	-	0.053	-	0.049	-
	C4	*Phormidium tenue*	-	0.052	-	-	-	-	-	-	-	-	-	0.064
	C5	*Microcystis* sp.	-	-	-	0.146	-	0.027	-	-	-	-	-	-
	C6	*Chroococcus limneticus*	-	-	-	-	-	0.051	-	-	-	-	-	-
	C7	*Merismopedia punctata*	-	-	-	-	-	0.058	-	-	-	-	-	-
	C8	*Merismopedia tenuissima*	-	-	-	-	-	-	-	-	-	0.105	-	0.092

**Table 3:** Similarity index of phytoplankton communities at each space of Dongting Lake.

Month (Hydrological Period)	Space	SK	SS	QJ	WDL	SDL	EDL
August (water connected)	SK	1.00	0.38	0.42	0.49	0.37	0.48
	SS	III	1.00	0.40	0.41	0.35	0.38
	QJ	III	III	1.00	0.31	0.42	0.59
	WDL	IV	III	0.31	1.00	0.40	0.30
	SDL	III	III	III	0.40	1.00	0.27
	EDL	III	III	IV	III	III	1.00
September (water disconnected)	SK	1.00	0.11	0.27	0.18	0.19	0.24
	SS	II	1.00	0.25	0.16	0.15	0.20
	QJ	III	II	1.00	0.17	0.20	0.34
	WDL	II	II	II	1.00	0.22	0.24
	SDL	II	II	II	0.22	1.00	0.24
	EDL	II	II	III	II	II	1.00

**Table 4:** Comparison of phytoplankton abundance and eutrophication levels between Dongting Lake and other freshwater lakes in the middle and lower reaches of the Yangtze River.

Lake	Month	Abundance (×10^4^ cells/L)	Shannon–Wiener Index	Eutrophication Levels (TLI Index)	Reference
DTL	August (2022a)	122.06	3.30	Light eutrophic (53.85)	This study
	September (2022a)	351.18	2.12	Mesotrophic (43.09)	This study
SK	August (2017a)	100.9	1.58	Light–middle eutrophic (50–63.66)	[49]
SS		291.8	2.39		
DTL		576.6	1.86		
DTL	September (2019a)	31.10	2.81	Mesotrophic (46.48)	[40]
DTL	1991a–2020a	10.0–95.7	-	Mesotrophic–light eutrophic (41.09–51.68)	[50]
Poyang	August (2017a)	1630–5756	3.95	-	[51]
Chaohu	October (2020a)	239	3.12	Light eutrophic (57.3)	[52]
Hongze	2015a–2016a	5350	-	Light eutrophic (58.07)	[53]

## Data Availability

Data are available on request to the authors.

## References

[1] Jeppesen E., Kronvang B., Olesen J.E., Audet J., Sondergaard M., Hoffmann C.C., Andersen H.E., Lauridsen T.L., Liboriussen L., Larsen S.E. (2011). Climate change effects on nitrogen loading from cultivated catchments in Europe: Implications for nitrogen retention, ecological state of lakes and adaptation. Hydrobiologia.

[2] Strayer D.L., Dudgeon D. (2010). Freshwater biodiversity conservation: Recent progress and future challenges. J. N. Am. Benthol. Soc..

[3] Iqbal M.M., Li L., Hussain S., Lee J.L., Mumtaz F., Elbeltagi A., Waqas M.S., Dilawar A. (2022). Analysis of seasonal variations in surface water quality over wet and dry regions. Water.

[4] Ayele H.S., Atlabachew M. (2021). Review of characterization, factors, impacts, and solutions of Lake eutrophication: Lesson for lake Tana, Ethiopia. Environ. Sci. Pollut. Res..

[5] Yu S., Yu G.B., Liu Y., Li G.L., Feng S., Wu S.C., Wong M.H. (2012). Urbanization impairs surface water quality: Eutrophication and metal stress in the grand canal of China. River Res. Appl..

[6] Iqbal M.M., Hussain S., Cheema M., Jehanzeb M., Lee J.L., Waqas M.S., Aslam M.A. (2022). Seasonal effect of agricultural pollutants on coastline environment: A case study of the southern estuarine water ecosystem of the boseong county Korea. Pak. J. Agri. Sci..

[7] Qiu J. (2010). China drought highlights future climate threats. Nature.

[8] Min S.K., Zhang X.B., Zwiers F.W., Hegerl G.C. (2011). Human contribution to more-intense precipitation extremes. Nature.

[9] Xia J., Chen J., She D. (2022). Impacts and countermeasures of extreme drought in the Yangtze River Basin in 2022. J. Hydraul. Eng..

[10] Zhang L.X., Zhou T.J. (2015). Drought over East Asia: A Review. J. Clim..

[11] Zhang W.J., Jin F.F., Zhao J.X., Qi L., Ren H.L. (2013). The Possible Influence of a Nonconventional El Nino on the Severe Autumn Drought of 2009 in Southwest China. J. Clim..

[12] Li S.Y., Bush R.T., Mao R., Xiong L.H., Ye C. (2017). Extreme drought causes distinct water acidification and eutrophication in the Lower Lakes (Lakes Alexandrina and Albert), Australia. J. Hydrol..

[13] Djabri L., Bouhsina S., Hani A., Bosch A., Mudry J., Djouamaa M.C. (2014). Impacts of drought on water quality: The case of aquifers in eastern Algeria. Evolving Water Resources Systems: Understanding, Predicting and Managing Water-Society Interactions.

[14] Barroso H.D., Tavares T.C.L., Soares M.D., Garcia T.M., Rozendo B., Vieira A.S.C., Viana P.B., Pontes T.M., Ferreira T.J.T., Pereira J. (2018). Intra-annual variability of phytoplankton biomass and nutrients in a tropical estuary during a severe drought. Estuar. Coast. Shelf Sci..

[15] Whitton B.A. (2012). Changing approaches to monitoring during the period of the ‘Use of Algae for Monitoring Rivers’ symposia. Hydrobiologia.

[16] Cai Y., Qi L., Shan T., Liu Y., Zhang N.N., Lu X.X., Fan Y.W. (2022). Application of Phytoplankton Taxonomic alpha-Diversity Indices to Assess Trophic States in Barrier Lake: A Case of Jingpo Lake. Diversity.

[17] McCarthy J.J., Goldman J.C. (1979). Nitrogenous nutrition of marine phytoplankton in nutrient-depleted waters. Science.

[18] Sunda W.G., Huntsman S.A. (1997). Interrelated influence of iron, light and cell size on marine phytoplankton growth. Nature.

[19] Yuan Y.X., Jiang M., Liu X.T., Yu H.X., Otte M.L., Ma C.X., Her Y.G. (2018). Environmental variables influencing phytoplankton communities in hydrologically connected aquatic habitats in the Lake Xingkai basin. Ecol. Indic..

[20] Anderson S.R., Harvey E.L. (2019). Seasonal variability and drivers of microzooplankton grazing and phytoplankton growth in a subtropical estuary. Front. Mar. Sci..

[21] Xu Y.Y., Cai Q.H., Ye L., Shao M.L. (2011). Asynchrony of spring phytoplankton response to temperature driver within a spatial heterogeneity bay of Three-Gorges Reservoir, China. Limnologica.

[22] Ward B.B., Van Oostende N. (2016). Phytoplankton assemblage during the North Atlantic spring bloom assessed from functional gene analysis. J. Plankton Res..

[23] Hrdinka T., Novicky O., Hanslik E., Rieder M. (2012). Possible impacts of floods and droughts on water quality. J. Hydro-Environ. Res..

[24] Worrall F., Burt T.P. (2008). The effect of severe drought on the dissolved organic carbon (DOC) concentration and flux from British rivers. J. Hydrol..

[25] Mosley L.M. (2015). Drought impacts on the water quality of freshwater systems; review and integration. Earth Sci. Rev..

[26] Wang W.J., Shi K., Wang X.W., Wang S.Q., Zhang D., Peng Y.Y., Li N., Zhang Y.L., Zhang Y.B., Qin B.Q. (2023). A record-breaking extreme heat event caused unprecedented warming of lakes in China. Sci. Bull..

[27] Yu Y.W., Mei X.F., Dai Z.J., Gao J.J., Li J.B., Wang J., Lou Y.Y. (2018). Hydromorphological processes of Dongting Lake in China between 1951 and 2014. J. Hydrol..

[28] Yan G.H., Yin X.Y., Huang M.S., Wang X., Huang D.Z., Li D. (2023). Dynamics of phytoplankton functional groups in river-connected lakes and the major influencing factors: A case study of Dongting Lake, China. Ecol. Indic..

[29] Han Q.Q., Zhang S.H., Huang G.X., Zhang R. (2016). Analysis of long-term water level variation in Dongting Lake, China. Water.

[30] Liang C., Li H.Q., Lei M.J., Du Q.Y. (2018). Dongting Lake Water Level Forecast and Its Relationship with the Three Gorges Dam Based on a Long Short-Term Memory Network. Water.

[31] Liu Y.Z., Jiang C.B., Long Y.N., Deng B., Jiang J.Y., Yang Y., Wu Z.Y. (2023). Study on the Water Level-Discharge Relationship Changes in Dongting Lake Outlet Section over 70 Years and the Impact of Yangtze River Backwater Effect. Water.

[32] Zhou H., Luo Z.C., Tangdamrongsub N., Wang L.C., He L.J., Xu C., Li Q. (2017). Characterizing Drought and Flood Events over the Yangtze River Basin Using the HUST-Grace2016 Solution and Ancillary Data. Remote Sens..

[33] Huang Q., Sun Z.D., Opp C., Lotz T., Jiang J.H., Lai X.J. (2014). Hydrological Drought at Dongting Lake: Its Detection, Characterization, and Challenges Associated With Three Gorges Dam in Central Yangtze, China. Water Resour. Manag..

[34] Ji H., Wu G., Liu Y. (2016). Sharp change of lake levels during the two extreme droughts and its hydroclimatic processes in Lake Dongting, China. J. Lake Sci..

[35] Zhang H., Wang Y.Y., Xu J. (2023). Influence of Seasonal Water Level Fluctuations on Food Web Structure of a Large Floodplain Lake in China. Sustainability.

[36] Xie Y.H., Chen X.S. (2008). Effects of Three-Gorge Project on Succession of Wetland Vegetation in Dongting Lake. Res. Agric. Mod..

[37] Yuan Y., Zeng G.M., Liang J., Huang L., Hua S.S., Li F., Zhu Y., Wu H.P., Liu J.Y., He X.X. (2015). Variation of water level in Dongting Lake over a 50-year period: Implications for the impacts of anthropogenic and climatic factors. J. Hydrol..

[38] Tian Z.B., Zheng B.H., Wang L.J., Li L.Q., Wang X., Li H., Norra S. (2017). Long term (1997–2014) spatial and temporal variations in nitrogen in Dongting Lake, China. PLoS ONE.

[39] Li F., Qin X.Y., Xie Y.H., Chen X.S., Hu J.Y., Liu Y.Y., Hou Z.Y. (2013). Physiological mechanisms for plant distribution pattern: Responses to flooding and drought in three wetland plants from Dongting Lake, China. Limnology.

[40] Yan G.H., Yin X.Y., Wang X., Huang M.S., Huang D.Z., Wang E.R., Zhang Y.Y. (2023). Driving factors of phytoplankton population and function group change in Dongting Lake and evaluation of water quality applicability. Chin. J. Environ. Sci..

[41] Ministry of Ecological Environment of the People’s Republic of China (2002). Methods for the Monitoring and Analysis of Water and Wastewater.

[42] Hu H.J., Wei Y.X. (2006). The Freshwater Algae of China: Systematics, Taxonomy and Ecology.

[43] Aksnes D.L., Wassmann P. (1993). Modeling the significance of zooplankton grazing for export production. Limnol. Oceanogr..

[44] Shannon C.E. (1963). The Mathematical Theory of Communication.

[45] Jaccard P. (1908). Nouvelles recherches sur la distribution florale. Bull. Soc. Vaud. Sci. Nat..

[46] Tan X., Xia X.L., Cheng X.L., Zhang Q. (2011). FTemporal and spatial pattern of phytoplankton community and its biodiversity indices in the Danjiangkou Reservoir. Chin. J. Environ. Sci..

[47] Hu J., Yang Y., Chi S., Shen Q., Hu J. (2017). Spatial variation of phytoplankton community structure and its relationship with environmental factors at the Mangshan pumping station. Acta Ecol. Sin..

[48] Lai J.S., Zou Y., Zhang J.L., Peres-Neto P.R. (2022). Generalizing hierarchical and variation partitioning in multiple regression and canonical analyses using the rdacca.hp R package. Methods Ecol. Evol..

[49] Wang M.Q., Wang J.C., Wang Q., Yang C.Y., Zou Z.H., Qian B. (2018). Characteristics of plankton community structure and eutrophication status in Dongting Lake in the season with normal water level. Chin. J. Ecol..

[50] Fu Z., Guo J., Huang D., Wang C. (2022). The evolution and influencing factors of eutrophication in Dongting Lake. Environ. Chem. Beijing China.

[51] Yang X., Ma J.S., Zhang H., Zhou Q. (2021). Community structure and the water quality during different hydrological periods in Poyang Lake. Acta Hydrobiol. Sin..

[52] Wu Z., Zhu C., Tang P., Yang X., Wang H., Zhang F. (2023). Correlation analysis of phytoplankton community and water quality factors in Chaohu Lake. J. Biol..

[53] Wu T., Liu J., Deng J., Dai X., Tang R., Peng K., Zou W., Cai Y., Gong Z. (2019). Community structure of phytoplankton and bioassessment of water quality in a large water-carrying lake, Lake Hongze. J. Lake Sci..

[54] Geng M.M., Wang K.L., Yang N., Li F., Zou Y.A., Chen X.S., Deng Z.M., Xie Y.H. (2021). Spatiotemporal water quality variations and their relationship with hydrological conditions in Dongting Lake after the operation of the Three Gorges Dam, China. J. Clean Prod..

[55] Guan X.W., Zeng M. (2022). Characteristics and enlightenment of low water in Changjiang River Basin in 2022. Yangtze River.

[56] Feng Y., Zheng B.H., Jia H.F., Peng J.Y., Zhou X.Y. (2021). Influence of social and economic development on water quality in Dongting Lake. Ecol. Indic..

[57] Rodrigues L.C., Simoes N.R., Bovo-Scomparin V.M., Jati S., Santana N.F., Roberto M.C., Train S. (2015). Phytoplankton alpha diversity as an indicator of environmental changes in a neotropical floodplain. Ecol. Indic..

[58] Connell J. (1978). Diversity in tropical rain forests and coral reefs. High diversity of trees and corals is maintained only in a nonequilibrium state. Science.

[59] Marengo J.A., Ambrizzi T., da Rocha R.P., Alves L.M., Cuadra S.V., Valverde M.C., Torres R.R., Santos D.C., Ferraz S.E.T. (2010). Future change of climate in South America in the late twenty-first century: Intercomparison of scenarios from three regional climate models. Clim. Dyn..

[60] Moss B., Kosten S., Meerhoff M., Battarbee R.W., Jeppesen E., Mazzeo N., Havens K., Lacerot G., Liu Z.W., De Meester L. (2011). Allied attack: Climate change and eutrophication. Inland Waters.

[61] Jeppesen E., Brucet S., Naselli-Flores L., Papastergiadou E., Stefanidis K., Noges T., Noges P., Attayde J.L., Zohary T., Coppens J. (2015). Ecological impacts of global warming and water abstraction on lakes and reservoirs due to changes in water level and related changes in salinity. Hydrobiologia.

[62] Bradbury J.P. (1975). Diatom Stratigraphy and Human Settlement in Minnesota.

[63] Liu J.F., Chen Y.W., Li M.J., Liu B.G., Liu X., Wu Z.S., Cai Y.J., Xu J.Y., Wang J.J. (2019). Water-level fluctuations are key for phytoplankton taxonomic communities and functional groups in Poyang Lake. Ecol. Indic..

[64] Yan X., Zhang Y., Li Y., Jiang Y., Cui Z., Gao X., Wu N., Nicola F., Han X. (2021). Hydrologic and physicochemical factors co-drive seasonal changes of phytoplankton during dynamic water diversion processes in the Danjiangkou Reservoir. J. Lake Sci..

[65] Jiang Z.B., Liu J.J., Chen J.F., Chen Q.Z., Yan X.J., Xuan J.L., Zeng J.N. (2014). Responses of summer phytoplankton community to drastic environmental changes in the Changjiang (Yangtze River) estuary during the past 50 years. Water Res..

[66] Till A., Rypel A.L., Bray A., Fey S.B. (2019). Fish die-offs are concurrent with thermal extremes in north temperate lakes. Nat. Clim. Chang..

[67] Walter K.M., Zimov S.A., Chanton J.P., Verbyla D., Chapin F.S. (2006). Methane bubbling from Siberian thaw lakes as a positive feedback to climate warming. Nature.

[68] de Emiliani M.O.G. (1997). Effects of water level fluctuations on phytoplankton in a river-floodplain lake system (Parana River, Argentina). Hydrobiologia.

[69] Liu X., Qian K.M., Tan G.L., Xing J.S., Li M., Chen Y.W. (2014). Phytoplankton Community Structure and Its Succession in Isolated Lakes of Poyang-Junshan Lake. Chin. J. Environ. Sci..

[70] Wang S.Y., Gao Y., Jia J.J., Kun S.D., Lyu S.X., Li Z.X., Lu Y., Wen X.F. (2021). Water level as the key controlling regulator associated with nutrient and gross primary productivity changes in a large floodplain-lake system (Lake Poyang), China. J. Hydrol..

[71] Istvanovics V., Honti M., Voros L., Kozma Z. (2010). Phytoplankton dynamics in relation to connectivity, flow dynamics and resource availability-the case of a large, lowland river, the Hungarian Tisza. Hydrobiologia.

[72] Wang S., Zhang X., Tian S., Cao Y., Chen R. (2020). Study on phytoplankton community structure characteristics and its influencing factors of lakes in arid regions of the Yellow River basin during ice-sealing period. J. Hydraul. Eng..

[73] Liu X., Li Y.A., Liu B.G., Qian K.M., Chen Y.W., Gao J.F. (2016). Cyanobacteria in the complex river-connected Poyang Lake: Horizontal distribution and transport. Hydrobiologia.

